# Clusterin overexpression in both malignant and nonmalignant prostate epithelial cells induces cell cycle arrest and apoptosis

**DOI:** 10.1038/sj.bjc.6602193

**Published:** 2004-10-19

**Authors:** M Scaltriti, S Bettuzzi, R M Sharrard, A Caporali, A E Caccamo, N J Maitland

**Affiliations:** 1 Dipartimento di Scienze Biomediche, Universita' di Modena e Reggio Emilia, Via GCampi, Modena 287 - 41100, Italy; 2 Dipartimento di Medicina Sperimentale, Plesso Biotecnologico Integrato, Universita'di Parma, Via Volturno, Parma 39 -43100, Italy; 3 YCR Cancer Research Unit, University of York, Heslington, York YO 10 5YW, UK

**Keywords:** clusterin, prostate, cell cycle, apoptosis, etoposide, IRES vector

## Abstract

Expression of the castration-induced clusterin protein is incompatible with the survival of human prostate cancer cells in tissues and in cell culture. To investigate the fate of human prostate epithelial cells, when engineered to maintain expression of clusterin protein, we have used an IRES-hyg vector and hygromycin selection. PC-3 prostate tumour cells were substantially more sensitive to clusterin expression than nonmalignant PNT1a cells, showing multiple phenotypic changes including cell cycle arrest and increased apoptosis. The results strengthen the hypothesis that clusterin expression is proapoptotic. Expression of exogenous clusterin in both cell types resulted in its relocation from the cytoplasm and a nuclear accumulation of the protein, as was also seen in the same cells when apoptosis was induced by etoposide treatment. To survive clusterin expression, the PC-3 tumour cells developed apoptosis-inhibitory properties. This could have significance for the resistance of prostate cancers to chemo/radiotherapy, where clusterin overexpression is observed.

Clusterin is a highly conserved heterodimeric-secreted glycoprotein present in most animal tissues and body fluids (Rosenberg and Silkensen, 1995). The protein has been implicated in several biological processes, but a definitive mechanism of action and role for this protein has not yet emerged. Clusterin is encoded by a single gene located on chromosome 8p21–p12, a locus commonly deleted in early stage prostate cancer (Macintosh *et al*, 1998). The protein precursor of 65–70 kDa is cleaved into two subunits, *α* and *β*, linked by five disulphide bonds. The three-dimensional arrangement of the subunits is currently unknown as no X-ray crystallographic data are available for clusterin (because of the ability of the protein to spontaneously aggregate in solution).

Since its first description in rat Sertoli cells in 1982 (Kissinger *et al*, 1982) and in ram rete testis fluid (Blaschuk *et al*, 1983), clusterin has been studied in several biological models, producing conflicting data. Subsequently, it was found to be the most potently induced gene in the rat ventral prostate after androgen ablation (Bettuzzi *et al*, 1989). Several functions have been proposed for clusterin, including chaperone activity (Lakins *et al*, 2002; Poon *et al*, 2003), complement inhibition, membrane protection (Silkensen *et al*, 1994), and recently, it has been described as a biomarker of senescence (Bettuzzi *et al*, 1994; Petropoulou *et al*, 2001), Alzheimer's disease (DeMattos *et al*, 2002), fertility (Ibrahim *et al*, 2000) and cancer prognosis (Bettuzzi *et al*, 2000; Miyake *et al*, 2002). Despite the involvement in a variety of pathologies, the role of clusterin in tumour progression and malignancy remains most controversial. Increased (Hough *et al*, 2001; Miyake *et al*, 2002) or decreased (Bettuzzi *et al*, 2000; Behrens *et al*, 2001; Zhang *et al*, 2003) expression was observed, depending on the organ or tissue studied. We have shown previously that prostate tumour tissue expresses significantly lower levels of clusterin compared to adjacent normal prostate tissue (Bettuzzi *et al*, 2000).

Equally controversial results have been obtained with regard to the role played by clusterin in apoptosis and cellular proliferation. Although several reports found no correlation between clusterin expression and cell proliferation (Petropoulou *et al*, 2001), and suggest a possible antiapoptotic role for this gene (Miyake *et al*, 2000; July *et al*, 2002), there is also evidence that clusterin can not only affect the cell cycle (Bettuzzi *et al*, 1999; Bettuzzi *et al*, 2002; Zhou *et al*, 2002), but also promote programmed cell death (Woolveridge *et al*, 2001; Han *et al*, 2003). In particular, a proapoptotic role for clusterin, facilitating the removal of severely damaged and genetically unstable cells, has also been suggested (Yang *et al*, 2000).

Generally, clusterin expression has been found to be upregulated in tissues undergoing regression, remodelling and apoptosis. Recently, a nuclear localisation of clusterin was suggested as a death signal (Yang *et al*, 1999, 2000) despite the presence of an *N*-terminal secretory tag. Nuclear translocation of the protein would derive from alternative splicing of clusterin mRNA, producing a shorter (5′-terminal truncated) messenger to produce the nuclear form (Leskov *et al*, 2003). Short-term apoptotic cytotoxicity, as well as a long-term clonogenic cytotoxicity response, emerges from these data, identifying clusterin as a death-inducing gene (Leskov *et al*, 2003).

Our previous work demonstrated that a transient overexpression of clusterin can affect the cell cycle in immortalised prostate epithelial cells by decreasing their proliferation rate (Bettuzzi *et al*, 2002). We now describe the production of stable clones of both SV40 immortalised normal prostate epithelial cells (PNT1a) and bone metastatic cells from prostate cancer (PC-3), which can overexpress clusterin. While the majority of cells die as a result of clusterin overexpression, rare survivors can be isolated. These cell clones have developed resistance to the apoptotic effects of clusterin.

## MATERIALS AND METHODS

### Cell lines

PNT1a (Cussenot *et al*, 1991) is a nontumorigenic epithelial cell line derived by SV40 immortalisation of normal prostate epithelium. PC-3 is a prostatic carcinoma cell line with an androgen-independent phenotype, purchased from the American Tissue Culture Collection. PNT1a cells were maintained in RPMI 1640 (Gibco, Paisley, UK) containing 2 mM glutamine and 10% fetal calf serum (FCS) (Gibco, Pasley, UK); PC-3 cells were maintained in Ham's F12 medium (ICN, Irvine, CA, USA) containing 2 mM glutamine and 7% FCS.

### Expression vectors

Full-length human clusterin cDNA was generated by RT–PCR from normal human fibroblast total RNA using the primers 5′-GACTCCAGAATTGGAGGCATG-3′ and 5′-ATCTCACTCCTCCCGGTGCT-3′ and cloned into pGEM T-easy (Promega, Southampton, UK).

From this vector, clusterin full-length cDNA was then subcloned into the bicistronic expression vector pIREShyg1 (Accession number U89672, Clontech, Oxford, UK) (Rees *et al*, 1996) to produce pIRES-clu. Empty pIREShyg1 was used for generating mock control clones (pIRES-hyg). Both expression vectors were completely sequenced before use.

### Transfection, selection of stable clones and etoposide treatment

A total of 5 × 10^5^ cells of each line were plated in 6-cm dishes and transfected using the nonliposomal reagent FuGENE 6 (Roche, Lewes, UK), according to the manufacturer's protocol. PNT1a and PC-3 cells were transfected with equimolar amounts of endotoxin-free preparations of pIRES-clu or pIRES-hyg (control). Transiently transfected cells were harvested 48 h after transfection.

Transfection efficiency was assessed by detection of green fluorescence protein (GFP) expression by fluorescence microscopy using the pEGFP-N1 vector (Clontech Laboratories, Inc., East Meadow Circle, Palo Alto, CA, USA) in parallel transfection experiments. Positively transfected cells were routinely more than 50% of the cell population.

For selection of stably transfected cell populations, hygromycin B (Roche, Lewes, UK) was added to the culture medium 48 h after transfection at a concentration of 50 *μ*g ml^−1^ for PC-3 and 200 *μ*g ml^−1^ for PNT1a cells. These concentrations were predetermined to give 100% cell kill within 10 days for each cell type.

Drug selection was maintained for 14 days, until visible individual colonies were detected, fixed in paraformaldehyde 4% and stained with Giemsa stain 0.4% (w v^−1^) in buffered methanol solution, pH 6.8, with stabilisers (Sigma, Dorset, UK).

Etoposide (Sigma, Dorset, UK) was added for 12 h, at the indicated doses, to parental PNT1a and PC-3 cells.

### Cell proliferation assay and fluorescence-activated cell sorter (FACS) analysis

Cell proliferation was studied using the cell proliferation reagent WST-1 (4-[3-(4-iodophenyl)-2-(4-nitrophenyl)-2H-5-tetrazolio]-1,3-benzene disulphonate) (Roche, Lewes, UK) according to the manufacturer's protocol. Briefly, the number of viable cells, seeded in triplicate in 96 well plates, was estimated on the basis of their ability to metabolise tetrazolium salt WST-1 to formazan by mitochondrial dehydrogenases. Quantification (OD) of the formazan dye, which directly correlates to the metabolically active number of cells, was carried on by a scanning microplate reader (ELISA reader) MRX II, Dinex Technology.

Cell cycle analysis was evaluated by FACS (Beckman coulter Epics® XL) after staining the samples with propidium iodide.

### Immunocytochemistry

Cells were fixed with 4% paraformaldehyde for 15 min, permeabilised with 70% ethanol for 15 min, washed in 50 mM Tris-HCl (pH 7.5)–150 mM NaCl (TBS), and blocked in blocking solution (1% casein blocking agent (Boehringer, Lewes, UK)–0.1% Tween 20 in TBS).

Staining was carried out with mouse monoclonal anti-hClusterin alpha chain, clone 41D (Upstate Biotechnology, Lake Placid, NY, USA) diluted 1 : 100 in blocking solution, followed by anti-mouse IgG–Alexa 488 conjugate (Molecular Probes, Leiden, The Netherlands) diluted 1 : 200 in blocking solution. Cells were then counter-stained with 1 *μ*g ml^−1^ 4′, 6-diamidino-2-phenylindole (DAPI, Sigma, Dorset, UK) or DRAQ5™ (Sigma, Dorset, UK) in TBS, washed three times in TBS and observed for fluorescence using a Nikon Eclipse fluorescent microscope. Images were captured using the Openlab program (Coventry UK).

To detect Annexin-V binding, the cultures were washed in TBS and incubated with Annexin-V–Alexa 568 (Boehringer, Lewes, UK) according to the manufacturer's protocol.

### Western blotting

Whole cells were resuspended in buffer containing 1% SDS and 1% dithiothreitol, heated to 100°C for 5 min, fractionated by 10% SDS–polyacrylamide gel electrophoresis and blotted to PVDF membranes (Immobilon P, Millipore, Watford, UK). Clusterin was detected with mouse monoclonal anti-hClusterin (see above). Procaspase-9, cleaved poly-(ADP-ribose) polymerase (PARP) and cleaved caspase-3 were detected with specific rabbit polyclonal IgGs (New England Biolabs, Hitchin, UK). Cytokeratins were detected with mouse monoclonal anti-pan-cytokeratin mixture (Sigma, Dorset, UK).

The membranes were incubated in blocking solution (as above) for 1 h, then in primary antibody for 1 h (clusterin) or overnight (procaspase-9, cleaved PARP and cleaved caspase-3). After 4 × 10 min washes in 0.1% Tween-20 in TBS (T-TBS), the membranes were incubated in anti-mouse or anti-rabbit-peroxidase (Sigma, Dorset, UK) diluted 1 : 5000 in blocking solution for 60 min. The blots were washed 4 × 15 min in T-TBS and detected by chemiluminescence (BM chemiluminescence substrate, Boehringer, Lewes, UK).

For protein staining Ponceau S solution 0.1% (w v^−1^) in 5% acetic acid (Sigma, Dorset, UK) was used.

## RESULTS

### Clonogenicity of clusterin overexpressing cells

**Figure 1:**
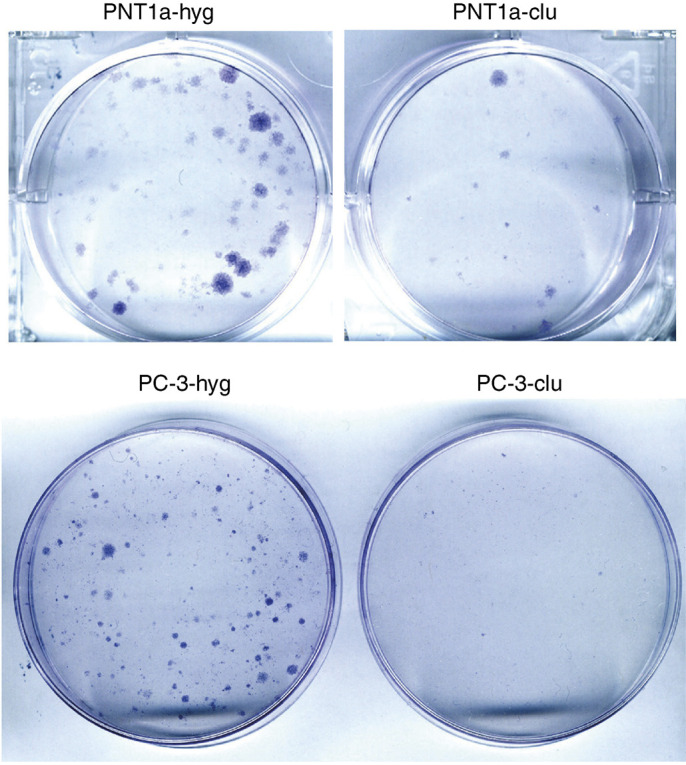
Colonies of hygromycin-resistant prostate epithelial cells expressing clusterin are smaller than those expressing only hyg^r^. Cell colonies selected in cell culture dishes, fixed with paraformaldehyde 4% and stained with Giemsa. Note the smaller size of the clu colonies (clu) compared to the hyg colonies (hyg), after 14 days of selection with hygromycin B. The figure shows a representative result obtained by performing the experiment four times using different standard endo-free plasmid preparations.

As shown in Figure 1

, PNT1a transfected with pIRES-clu, compared with PNT1a transfected with pIRES-hyg, resulted in a significantly lower number of hygromycin-resistant colonies. The pIRES-clu colony sizes were also generally smaller in diameter.

In transfected PC-3 cells, where drug selection was conducted with 50 *μ*g ml^−1^ of hygromycin B, clusterin overexpression produced more dramatic effects in terms of clonogenicity, since both the number and the size of the derived colonies were drastically reduced in comparison with the pIRES-hyg controls (Figure 1). The percentage reduction of clonogenicity in cells transfected with pIRES-clu was about 75% for PNT1a and 95% for PC-3.

### Characterisation of the stable clones

**Figure 2:**
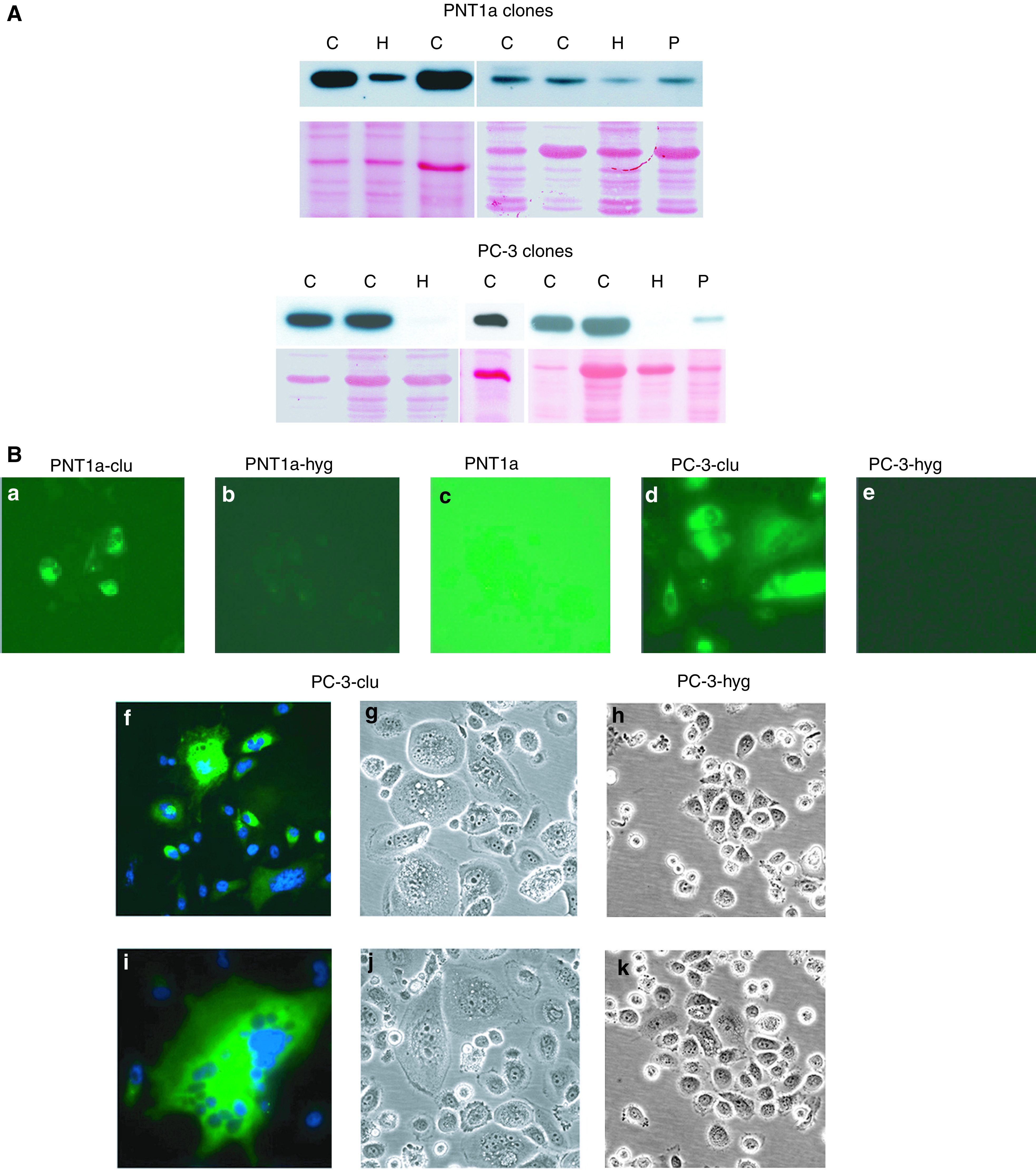
Clusterin expression patterns in stable clones of PNT1a and PC-3. (**A**) Western blot detection. The upper panel, for each cell type, shows clusterin detection in stable in clu clones (C) compared with hyg^r^ controls (H) and in parental cells (P). The lower panel provides an indication of total proteins transferred to the PVDF membrane by Ponceau staining. (**B**) Immunocytochemical detection. Panels a–e show Alexa-green fluorescence to visualise clusterin expression. Panel c (parental PNT1a) has been deliberately overexposed to reveal the weak cytoplasmic staining. Panels f and i show giant cells from two different PC-3-clu clones actively expressing clusterin (green stain), note the multiple nuclei, counterstained by DAPI (blue stain). A phase-contrast image of the giant cells is shown in panels g and j. Phase-contrast images of two different control PC-3 clones are shown in panels h and k. Magnification: × 400.

Individual colonies derived from PNT1a and PC-3, transfected with both pIRES-clu (PNT1a-clu) and pIRES-hyg (PNT1a-hyg), were isolated and expanded in the appropriate selection medium to allow the measurement of clusterin levels and intracellular distribution by Western blot (Figure 2A

) and immunocytochemistry (Figure 2B). Some distinctive morphological features were noted in the cells overexpressing exogenous clusterin. In particular, PNT1a-clu cells tolerated different levels of vector-expressed clusterin protein without any important morphological changes, whereas surviving PC-3-clu clones were entirely resistant to clusterin overexpression. Western blotting confirmed high levels of intracellular clusterin (Figure 2A) and a diverse range of clusterin expression levels in the individual clones.

The endogenous levels of clusterin expression in both PNT1a and PC-3 were relatively low by both Western blotting (Figure 2A, lanes P) and immunocytochemistry (Figure 2B), although the two cell lines were chosen as they represented the situation in normal and malignant tissues. As in prostate tissues, an immunohistochemical signal was barely detectable in PNT1a, showing a cytosolic localisation, and was normally undetectable in PC-3.

In all of the PNT1a-clu clones, expression of exogenous clusterin was significantly higher compared to the endogenous clusterin levels in the controls (PNT1a-hyg), but differences between the single clusterin clones were observed. No predictable morphological changes correlated with these differences and, as described below, no functional effects could be correlated to the variable clusterin expression levels. Owing to the severe cytotoxicity, PC-3 clusterin clones (PC-3-clu) were expected to express relatively low levels of this protein. Surprisingly, all five characterised PC-3-clu clones showed a similarly strong clusterin expression compared to the controls (PC-3-hyg), independently of the selection pressure (Figure 2).

Despite the endogenous low levels of this protein and the extensively limited tolerance to its overexpression exhibited during drug selection, PC-3-clu clones were nevertheless able to survive in the presence of large amounts of clusterin (Figure 2). During selection, PC-3-clu cells underwent several rounds of karyokinesis but were unable to overcome the G2–M phase, resulting in giant cells that were neither senescent nor apoptotic while maintaining high levels of clusterin expression. The stable PC-3-hyg clones looked like normal PC-3 cells (data not shown).

### Proliferation analysis of the stable clones

**Figure 3:**
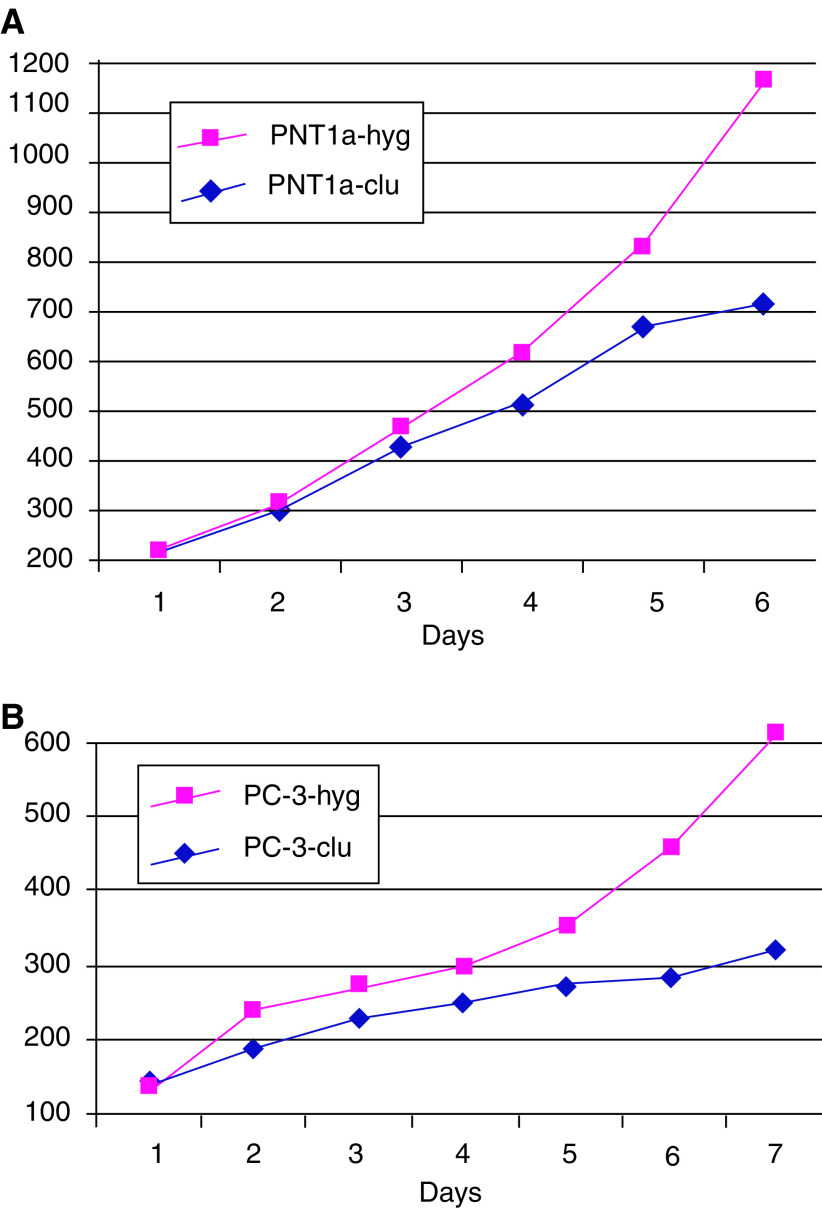
WST-1 proliferation analysis of representative stably transformed clones. (**A**) Comparative proliferation rates of two representative stably transformed PNT1a-clones. Red: PNT1a-hyg (from two analysed); blue: PNT1a-clu (from four analysed). (**B**) Comparative proliferation rates of PC-3 clones. Red: PC-3-hyg (from two analysed); blue: PC-3-clu (from two analysed). *Y*-axis: OD at 450 nm values correlate with the viable cell numbers.

**Figure 4:**
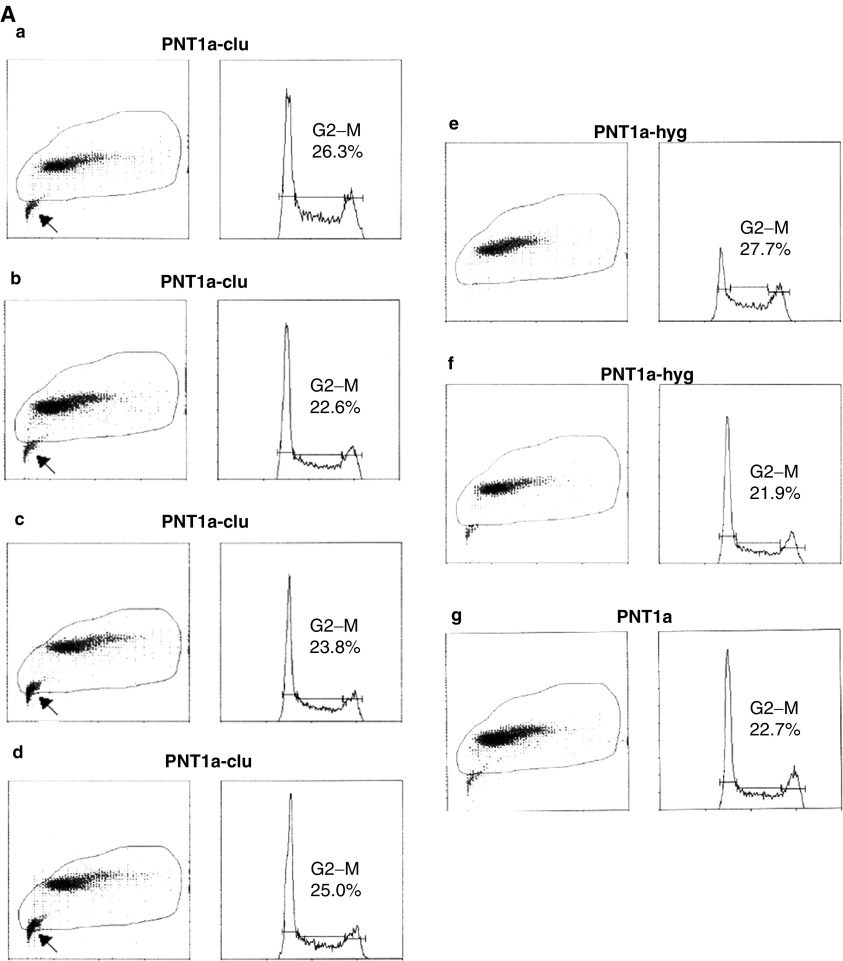

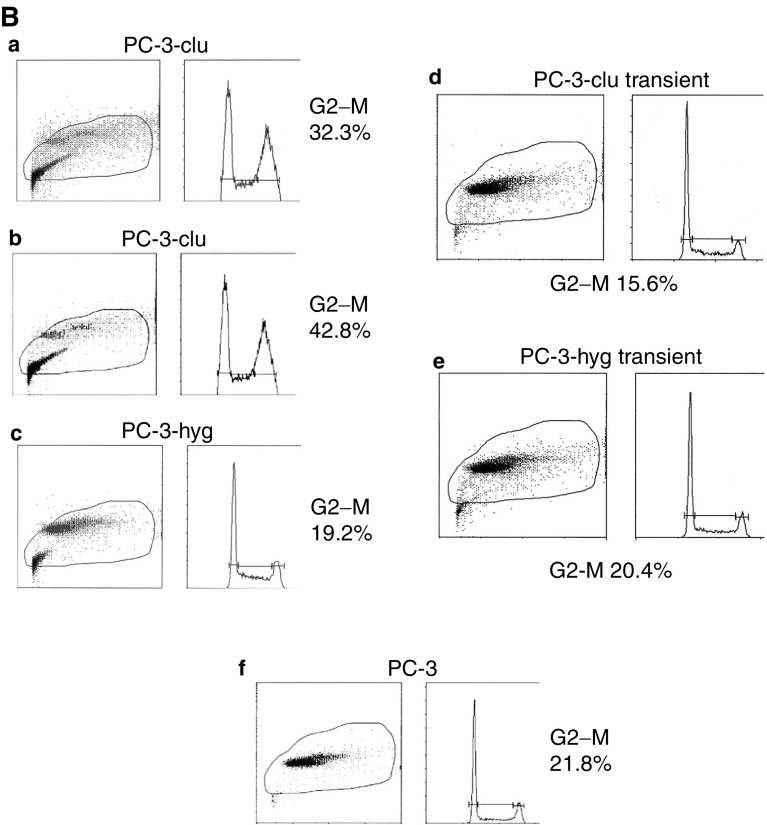
FACS analysis of stable and transient transfectants. (**A**) Cell cycle analysis of stable PNT-clu (four independent clones, a–d) and PNT-hyg (two independent clones, e and f) in comparison with PNT1a parental cells (g). G2/M proportions are indicated for each cell type. Arrows indicate the presence of increased (apoptotic) cellular debris in all four PNT1a-clu populations relative to PNT1a-hyg and parental PNT1a cells. (**B**) Cell cycle analysis of stable PC-3-clu (two independent clones, a and b) and stable PC-3-hyg (c) in comparison with parental PC-3 cells (f) and PC-3 cells transiently transfected with pIRES-clu (d) and pIRES-hyg (e). G2/M proportions are indicated for each cell type.

After cloning and 6 days of culture, the average number of PNT1a-clu cells as assessed by WST-1 assay (to overcome problems associated with counting multinucleate cells) was significantly lower than PNT1a-hyg cells, showing a decrease of 50% (Figure 3A

). To further investigate whether this phenomenon was due to blocked cell cycle or increasing cell progression or increasing cell death, a monoparametric analysis by fluorescence-activated cell sorter for each clone was performed (Figure 4A

). The data provide evidence of an increased apoptotic fraction (arrowed) in four independent PNT1a-clu clones compared with both PNT1a-hyg clones and PNT1a parental cells. Viable PNT1a-clu cells did not show a significant variation of the cell cycle phases compared to both controls and parental PNT1a cells, suggesting that lower growth rate observed was very likely due only to increased cell death.

For PC-3 cells, significant differences in viability/metabolic capacity were evident when compared with controls (Figure 3B). Fluorescence-activated cell sorter analysis of the stable PC-3-clu clones indicated a two-fold rise in the G2/M fraction, while PC-3-hyg clones had a cell cycle profile that was indistinguishable from parental PC-3 cells. In contrast, transient transfection of PC-3 with the same clusterin expression plasmid (Figure 4B) resulted in a 25% decrease of the G2–M phase, as we have previously reported in transiently transfected PNT1a cells (Bettuzzi *et al*, 2002).

### Intracellular location of clusterin under apoptotic conditions

**Figure 5:**
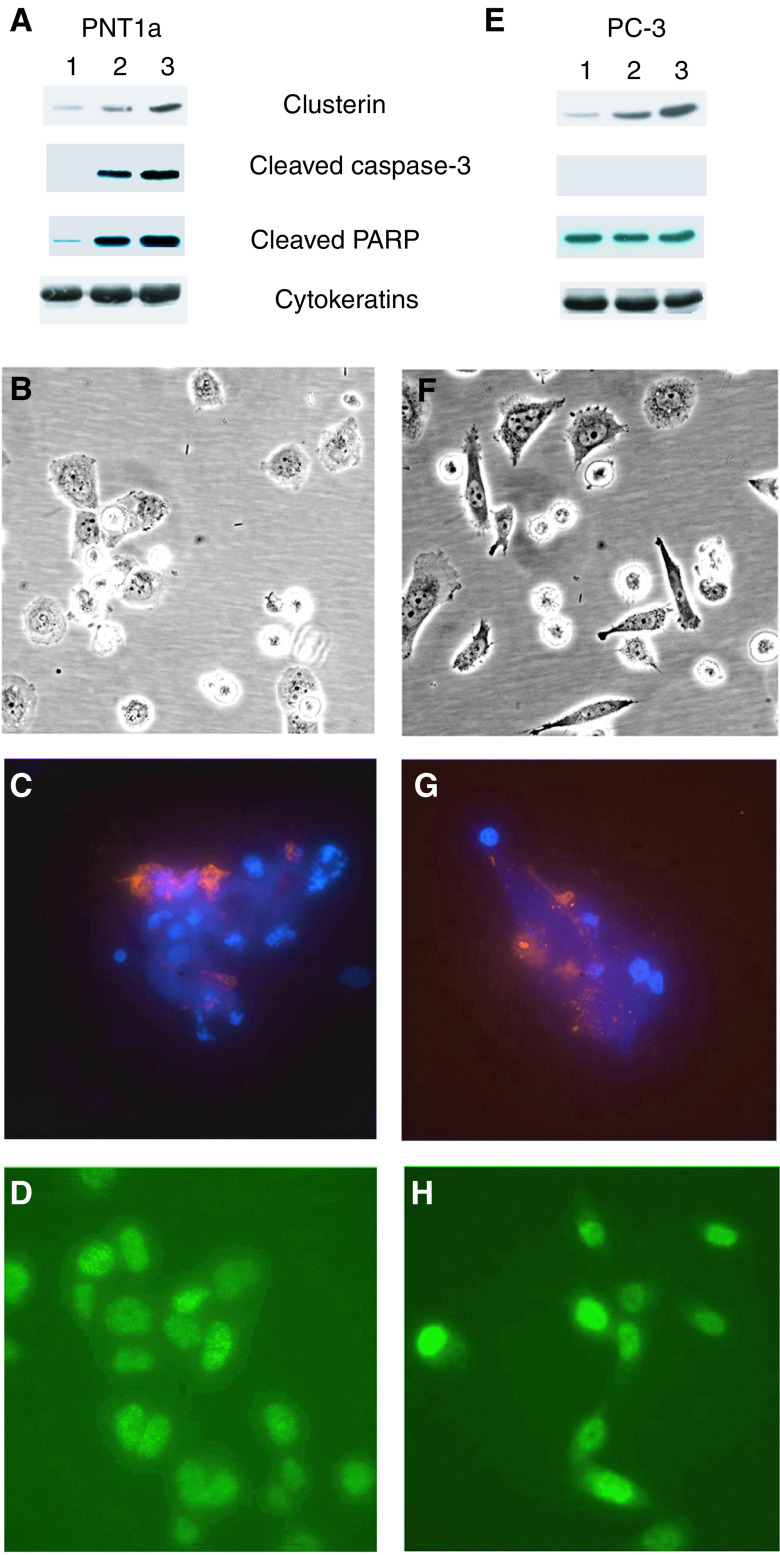
Etoposide-mediated effects on clusterin expression in PNT1a and PC-3 cells. Western blot detection of clusterin expression, cleaved caspase-3 and cleaved PARP in PNT1a (**A**) and PC-3 (**E**) cells. Equal protein loading was confirmed by pan-cytokeratin detection. Lanes 1: parental untransfected cells; lanes 2: parental cells treated overnight with 200 *μ*M etoposide; lanes 3: parental cells treated overnight with 400 *μ*M etoposide. PNT1a and PC-3 treated overnight with 400 *μ*M etoposide (phase images **B** and **F**) were tested for Annexin-V staining (respectively, **C** and **G**) and clusterin immunostaining (respectively, **D** and **H**). Magnification: × 400.

The data presented above suggest that stable overexpression of clusterin was sufficient to cause a decreased cell proliferation and an increase in cell death for both PNT1a and PC-3. This was accompanied by a nuclear translocation of the protein. To test this hypothesis, we further investigated clusterin expression and its localisation in response to chemical induction of apoptosis in these two cell lines. Etoposide is a well-known topoisomerase II inhibitor, widely used in cell biology to study apoptosis (Burden *et al*, 1996). Treatment of both PNT1a and PC-3 cells with several different concentrations of etoposide results within 12 h in a strong induction of apoptosis as assessed by Annexin-V binding, and a marked increase of clusterin expression (Figure 5

).

Intriguingly, we found considerable differences between these two cell lines in terms of the activation of apoptotic pathways in response to the DNA breaks induced by etoposide. Indeed, PNT1a cells showed a marked caspase-3 cleavage and subsequent PARP activation, while apoptosis in PC-3 cells was apparently caspase-independent (Figure 5). By immunocytochemistry (Figure 5, panels d and h), a clear nuclear localisation of clusterin was observed in virtually every cell after etoposide treatment. Therefore, expression of clusterin and its migration to the nucleus appeared to be one of the earliest events in PNT1a and PC-3 cells undergoing apoptosis.

### Molecular features of apoptosis in response to stabilised clusterin expression

**Figure 6:**
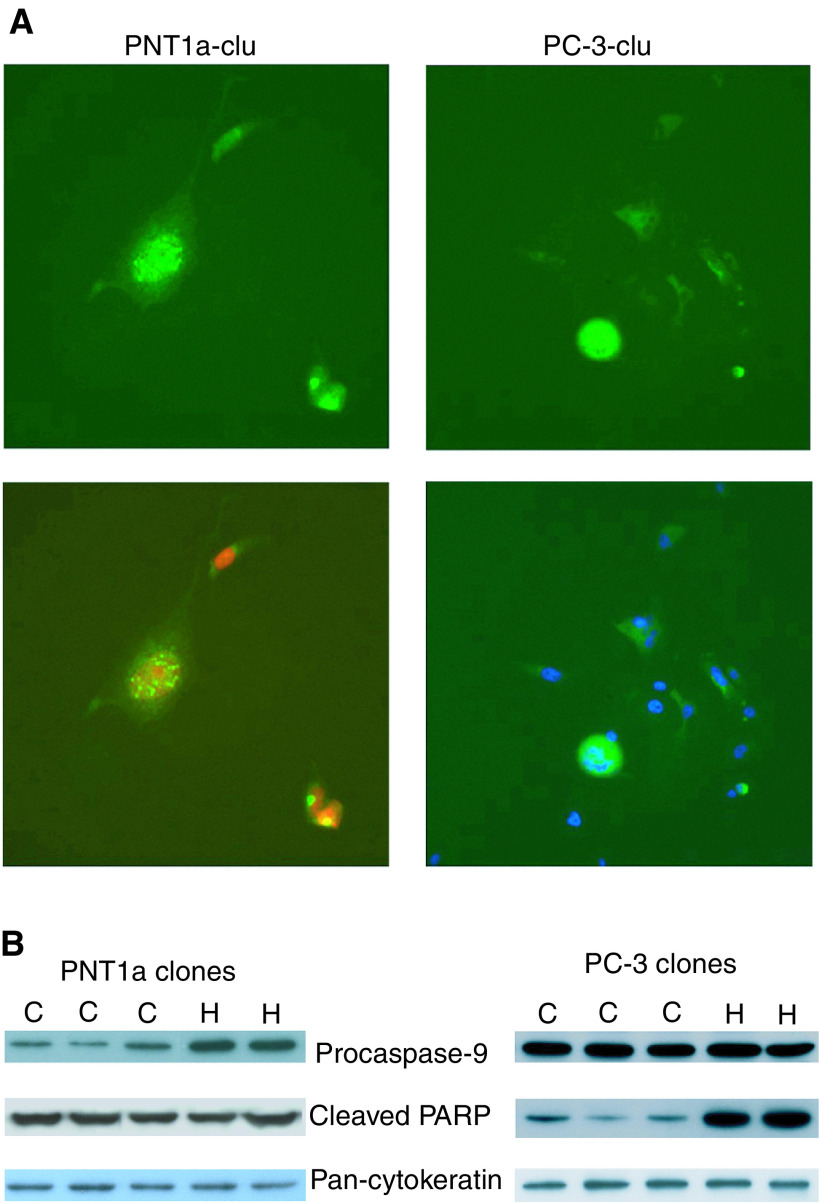
Phenotype of PNT1A and PC-3 cells expressing clusterin.(**A**) Alexa-green fluorescence visualisation of clusterin localisation in PNT1a-clu cells (upper left) and PC-3-clu cells (upper right). The same images are shown, merged with DRAQ 5 red counterstaining (lower left) and with DAPI blue counterstaining (lower right). Magnification: × 400. (**B**) Procaspase-9 and cleaved PARP detected by Western blot in PNT1a-clu and PC-3-clu clones (C) as compared to PNT1a-hyg and PC-3-hyg clones (H). Equal protein loading was confirmed by pan-cytokeratin detection.

To assess apoptotic cascades in the PNT1a and PC-3 stable clones, the expression of procaspase-9, cleaved caspase-3 and cleaved PARP were measured by Western blot. In both PNT1a and PC-3-clu clones, cleaved caspase-3 was undetectable. Between PNT1a-clu clones, although no differences in cleavage of PARP were observed, a decrease of procaspase-9 was however detected, while the PC-3-clu clones did not reveal significant variations of these two markers when compared to the controls (Figure 6B

).

Cleavage of PARP has been shown to be a valid marker for apoptosis in PC-3 cells (Chinni *et al*, 2001; Voelkel-Johnson *et al*, 2002). In PC-3-clu cells, a significant reduction of cleaved PARP was found relative to PC-3-hyg (Figure 6B) showing a possible mechanism of resistance to apoptosis.

## DISCUSSION

Previous studies carried out in nonmalignant PNT1a cells have demonstrated that a transient overexpression of clusterin is able to slow down the cell cycle (Bettuzzi *et al*, 2002). Since endogenous clusterin can be (barely) detected in the PNT1a cells, we sought to reproduce this result in PC-3 cells, malignant prostate epithelial cells in which endogenous clusterin is almost undetectable by Western blot. Transient expression of clusterin in PC-3 cells confirmed a cell cycle arrest, but distinct from that observed in PNT1a (Bettuzzi *et al*, 2002). No clearly classical activation of apoptotic pathways was recognised in PC-3 under this temporary high level of clusterin expression.

More homogeneous levels of clusterin expression within a cell can be achieved by establishment of stable cell lines. However, with both prostate cell lines, the generation of permanent cell lines engineered to overexpress clusterin proved difficult. By employing the IRES strategy in which drug resistance is directly linked to the expression of the exogenous clusterin, a number of rare, independently derived clusterin expressing clones were isolated from both cell lines. We have previously employed this strategy to examine the biological consequences of PTEN expression in these cell types (Sharrard and Maitland, 2000). There was a significantly higher yield of clones from PNT1a cells, which express higher levels of endogenous clusterin, compared to PC-3 cells. However, as shown in Figure 3, slower population doubling times were a common characteristic of the clusterin transformants, but essential morphological differences were found between PC-3 and PNT1a clones.

PC-3-clu cells maintained the features developed during selection. PNT1a-clu clones did not exhibit any significant morphological difference compared to the controls and maintained signs of functional apoptotic pathways. The differences shown by PC-3 cells, relative to PNT1a, could be due to the more aggressive phenotype of androgen-independent PC-3 cells, which would make apoptosis induction more difficult, whereas PNT1a cells, although immortalised with large T of SV40, are more differentiated and not tumorigenic (Cussenot *et al*, 1991).

Paradoxically, the PNT1a-clu clones, despite higher levels of the endogenous protein and the higher colony-forming efficiency, revealed relatively lower clusterin expression compared with PC-3-clu cells. This apparent contradiction could be explained by a form of resistance acquired by PC-3-clu survivors to the high levels of clusterin. One distinction between PC-3-clu and PNT1a-clu cells is highlighted in Figure 6 where, in PNT1a-clu clones, the relatively low level of procaspase-9, together with the data obtained by FACS analysis (Figure 4A), suggest that a limited apoptotic activity in PNT1a-clu clones has been maintained. PC-3-clu cells, on the contrary, required a precise mechanism to escape apoptosis, and a capacity to by-pass PARP cleavage may explain survival in the presence of elevated levels of clusterin. These data are apparently in contrast with the report by Zellweger *et al* (2003), who recently described an extended protection against radiation-induced cell death in stable transfected LNCaP cells overexpressing clusterin. Zellweger *et al* hypothesised that upregulation of this protein can represent an adaptive cell-survival mechanism on the basis of their previous observation that clusterin is overexpressed in prostate cancer (Miyake *et al*, 2000). This observation was not confirmed by our (Bettuzzi *et al*, 2000; Scaltriti *et al*, 2004; Caporali *et al*, 2004) and other publications (Stuart *et al*, 2004), demonstrating that clusterin is indeed more frequently *downregulated* in prostate cancer. Moreover, we have provided evidence to show that only PC-3 cells surviving to clonogenic selection, and thus exhibiting an apoptosis-resistance phenotype, can tolerate high levels of clusterin expression. Accordingly, the acquisition of resistance to apoptosis by PC-3 cells can be envisaged as an event that is necessary to survive clusterin overexpression, and not the opposite.

Thus, an alternative explanation for the same phenomenon could be that the apoptosis-resistance features acquired by PC-3-clu cells are consequent to previous clonogenic selection, and therefore these clones are the result of clusterin overexpression-escaped cell death.

A further variation of clusterin expression pattern in clu clones was detected by immunocytochemistry, where the protein was found to be located, in contrast to the normal cytoplasmic localisation, in both the nucleus and cytoplasm (Figures 2 and 6A). Recently, a nuclear form of clusterin, which lacks the secretion signal as a result of an alternative splicing, was described by Leskov *et al* (2003) after treating cells with ionising radiation. This particular form becomes specifically located in the cell nucleus, and is responsible for cell death. However, in the presence of high intracellular clusterin expression, the protein (which contains at least two NLS) could enter into the nucleus even without a requirement for splicing. Therefore, the nuclear relocalisation of clusterin in both PNT1a-clu and PC-3-clu clones was not unexpected. By silencing the expression of clusterin gene using small interfering RNA (siRNA), Trougakos *et al* (2004) showed that the selective knock down of the secreted form of this protein induced apoptosis in PC-3 cells. Surprisingly, the siRNA approach did not exert significant effects on intracellular clusterin. This finding indirectly suggests that clusterin might act differently depending on its subcellular localisation. Indeed, in a more recent work, the same authors reported cytotoxic properties of this protein when accumulated in high amounts intracellularly (Trougakos and Gonos, 2004).

To further establish the link between apoptosis and intracellular clusterin location, we carried out the inverse experiment to localise clusterin expression within both PNT1a and PC-3 cells, after etoposide-induced apoptosis. After 12 h of etoposide treatment, increased clusterin expression was observed in a dose-dependent manner, in both PNT1a and PC-3 cells (Figure 5) by both Western blot (Figure 5A and E) and immunocytochemistry (Figure 5D and H). Etoposide treatment therefore not only upregulates clusterin expression but also drives its localisation into the nucleus of virtually all of the treated cells. This correlates with an increase in cell rounding, detection of Annexin-V and, in PNT1a cells, activation and cleavage of caspase-3 and PARP.

Taken together, the etoposide and stable clusterin overexpression data would suggest that clusterin translocation into the cell nucleus, where it can interact with gene products involved in cell cycle regulation, DNA repair (Yang *et al*, 1999) and/or programmed cell death, plays a central role in its biological activity (Yang *et al*, 2000; Leskov *et al*, 2003). An analysis of the expression levels of cell cycle-related proteins such as cyclin A, cyclin B and p27 by Western blot after manipulation of clusterin levels was, however, inconclusive.

We conclude that, although the PNT1a and PC-3 cells respond differently to clusterin overexpression, this protein primarily affects cell cycle progression while activating different apoptotic pathways according to cell type, including a caspase-3-independent mechanism (Han *et al*, 2003). However, in PC-3 cells, which express only minimal levels of endogenous clusterin, precise apoptosis inhibitory mechanisms, such as resistance to cleavage of PARP, are required to overcome this phenomenon. Such antiapoptotic mechanisms may provide the basis for prostate tumour resistance to many conventional therapies. Further study of the clusterin-resistant PC-3 cells and a comparison to wild type should provide further evidence of the molecular basis of tumour resistance to apoptosis induction.
